# Characterization of WWOX expression and function in canine mast cell tumors and malignant mast cell lines

**DOI:** 10.1186/s12917-020-02638-3

**Published:** 2020-10-31

**Authors:** Rebecca Makii, Hanna Cook, Darian Louke, Justin Breitbach, Ryan Jennings, Christopher Premanandan, Eric M. Green, Joelle M. Fenger

**Affiliations:** 1 Department of Veterinary Clinical Sciences, College of Veterinary Medicine, The Ohio State University, 1900 Coffey Road, 444 Veterinary Medical Academic Building, Columbus, OH USA; 2 Department of Veterinary Biosciences, College of Veterinary Medicine, The Ohio State University, Columbus, OH USA

**Keywords:** Canine mast cell tumors, WWOX, DNA damage response

## Abstract

**Background:**

The WW domain-containing oxidoreductase (WWOX) tumor suppressor gene is frequently lost in a variety of solid and hematopoietic malignancies in humans. Dysregulation of WWOX has been implicated as playing a key role in tumor cell survival, DNA damage repair, and genomic stability. The purpose of this study was to characterize WWOX expression in spontaneous canine mast cell tumors (MCTs) and malignant cell lines and investigate the potential contribution of WWOX loss on malignant mast cell behavior.

**Methods/results:**

WWOX expression is decreased in primary canine MCTs and malignant mast cell lines compared to normal canine bone marrow-cultured mast cells. In transformed canine mastocytoma cell lines, overexpression of WWOX or WWOX knockdown had no effect on mast cell viability. Inhibition of WWOX enhanced clonogenic survival following treatment with ionizing radiation in the C2 mast cell line. Lastly, immunohistochemistry for WWOX was performed using a canine MCT tissue microarray, demonstrating that WWOX staining intensity and percent of cells staining for WWOX is decreased in high-grade MCTs compared to low-grade MCTs.

**Conclusions:**

These data suggest that WWOX expression is attenuated or lost in primary canine MCTs and malignant mast cell lines. Given the observed increase in clonogenic survival in WWOX-deficient C2 mast cells treated with ionizing radiation, further investigation of WWOX and its role in mediating the DNA damage response in malignant mast cells is warranted.

**Supplementary Information:**

**Supplementary information** accompanies this paper at 10.1186/s12917-020-02638-3.

## Background

Mast cell tumors (MCTs) are the most common malignant skin tumor in dogs, accounting for approximately 7–20% of all cutaneous tumors [1–5]. The biological behavior of canine MCTs is extremely variable, ranging from solitary, benign tumors to extremely aggressive tumors that metastasize to locoregional lymph nodes and distant organ sites. Several prognostic factors, including clinical stage and histopathologic grade using a 3- or 2-tier grading system aid in the classification of canine MCTs; however, the prognostic significance of histologic grade is associated with survival time and may not accurately predict metastasis. To this end, 37.5% of MCTs classified as ‘low grade’ using the Kiupel histologic grading system were from dogs with distant metastatic disease and 21.9% of ‘high grade’ MCTs were from dogs without evidence of distant metastases [6].

The etiology of canine MCTs is largely unknown; however, the identification of activating mutations in the proto-oncogene *c-KIT* in approximately 30% of dogs with aggressive MCTs has provided insight into the genetic changes that mediate the biological behavior of MCTs [7–10]. It has also resulted in the successful development and approval of a novel targeted therapeutic, Toceranib phosphate (Palladia®) that works primarily by inhibiting KIT signaling [11]. While data suggests that KIT inhibitors have significant biologic efficacy in the setting of KIT mutation, responses are generally not durable beyond 12 months and treatment is often unsuccessful in the ~ 70% of dogs that do not possess KIT mutations [7, 8]. While the role of KIT dysfunction in mast cell neoplasia has been well described, a more complete understanding of the additional molecular factors that influence malignant mast cell behavior is necessary to more effectively identify novel targets for therapeutic intervention. To this end, recent genome-wide gene expression analyses suggest that the presence of distinct subclasses of low- and high-risk MCTs exist with respect to their underlying molecular phenotypes and prognoses [12, 13]. These include enrichment of factors associated with proliferation pathways and overexpression of genes associated with the extracellular matrix that are linked to the activity of cancer-associated fibroblasts present in high-risk MCT stroma. Similarly, genome-wide DNA copy number analyses demonstrate that recurrent DNA copy number aberrations (CNAs) are associated with KIT mutation status and high histological grade, suggesting that loss or gain of genes within copy number aberrant regions may contribute to the neoplastic transformation of mast cells [14].

The WW domain-containing oxidoreductase (WWOX) is a highly conserved, 46 kDa protein consisting of two N-terminal WW domains and a C-terminal short-chain dehydrogenase/reductase domain [15]. The first WW-domain (WW1) is involved in protein-protein interactions by binding to partner proteins harboring proline-rich PPxY motifs and acts as an adaptor protein regulating their localization, transactivation, and stability, thereby influencing normal physiology and development [16–18]. Given its role in cellular metabolism, cell cycle checkpoint activation, and maintenance of genomic stability, dysregulation of WWOX has been implicated in cancer initiation and progression [19–21]. Initial evidence of the role of WWOX in tumor biology came from the discovery that the human *WWOX* gene resides in the genomic region FRA16D at 16q23, the second most common human chromosomal fragile site that is prone to frequent chromosome breakage [22, 23]. These “fragile sites” describe regions of recurrent chromosomal perturbations in the form of gaps, breaks, and rearrangements at specific DNA loci on metaphase chromosomes [23, 24]. The intriguing discovery that common fragile sites (CFSs) are late replicating regions known to be preferential hotspots for metaphase chromosome breaks and rearrangements led to scientific efforts to investigate the biological functions of *WWOX* and study its relevance in human health and disease.

There is now substantial data supporting the role of WWOX in cancer biology, including the observation that loss or attenuation of WWOX occurs in a number of human malignancies including bone, breast, lung, liver, ovarian, pancreatic, and many hematopoietic cancers [17, 25–28]. The genomic location of *WWOX* makes the locus susceptible to loss of heterozygosity (LOH) or homozygous deletion of the gene, resulting in reduced gene expression [20]. Alternatively, other epigenetic and genetic factors play a role in silencing WWOX expression. For example, hypermethylation of CpG islands at the *WWOX* promoter has been shown to transcriptionally inactivate WWOX in ovarian, pancreatic, lung, and breast cancer cell lines [28–32]. In contrast, the MCF7 breast cancer cell line does not show evidence of CpG methylation in the *WWOX* gene promoter and aberrant splicing events result in atypical nuclear localization and attenuated WWOX function [21].

Importantly, in vivo studies investigating the phenotypic consequences of global *Wwox* deletion found an increased incidence of spontaneous tumor formation in mice, suggesting a critical role for WWOX in tumorigenesis [19]. Additionally, heterozygous *Wwox (+/−*) mice developed significantly more ethyl nitrosourea-induced lung tumors and lymphoma compared to wild-type littermate mice, suggesting that *Wwox* haploinsufficiency is cancer predisposing. The versatile nature of WWOX is attributed, in part, to its ability to directly interact with proteins in a variety of cellular processes, including cell differentiation and growth. One of the first binding partners identified to interact with WWOX was p73, a p53 family homolog that mediates cellular response to stress, including regulating cell cycle checkpoint activation and apoptosis [33]. Loss of WWOX contributes to tumor progression and chemotherapeutic resistance through its interaction with several binding partners, including AP-2γ, STAT3, and ErbB4 among others [34–36]. Given the prevalence of WWOX dysregulation in cancer and its multifaceted role in regulating cell cycle progression, cellular metabolism, and DNA damage repair responses, WWOX represents a potential target for therapeutic intervention [33, 35, 37–41].

It is well established that WWOX expression is altered in many human malignancies and that WWOX functions as a tumor suppressor gene through dysregulation of target binding partners [19, 20]. Currently there is limited information regarding the potential role of WWOX dysregulation in malignant mast cell disease. As such, we sought to investigate the potential role of WWOX dysregulation in the biological behavior of canine MCTs. The purpose of this study was to characterize the expression of WWOX in canine primary MCT samples and malignant mast cell lines and to determine the functional consequences of altered WWOX expression in canine mast cell lines.

## Results

### WWOX expression is frequently decreased in primary canine MCT tissues and malignant mast cell lines

**Fig. 1:**
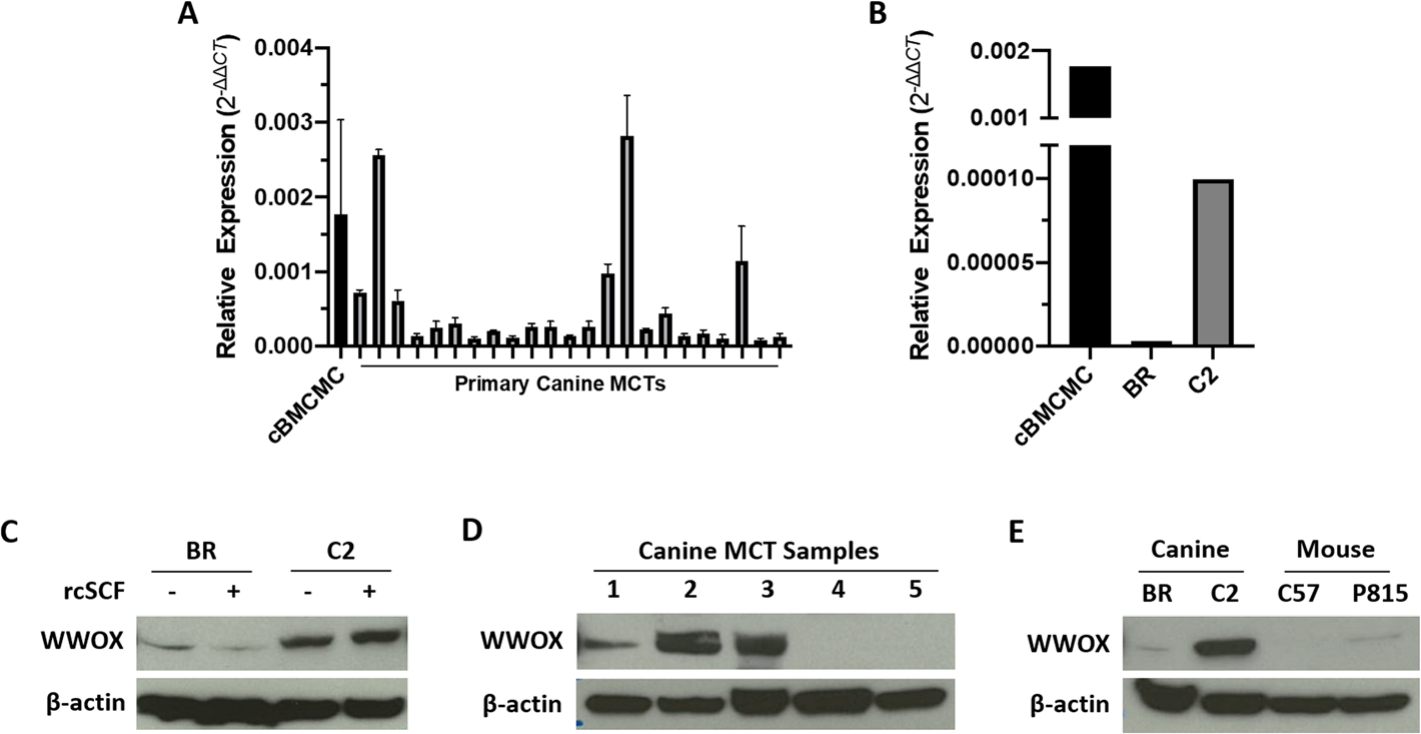
WWOX expression is reduced in canine MCTs and malignant mast cell lines. RNA was collected from **(a)** normal canine BMCMCs and fresh frozen canine MCT tissues and **(b)** malignant canine BR and C2 mast cell lines and quantitative RT-PCR for WWOX was performed. **c** Canine BR and C2 mast cell lines were left untreated or incubated with 100 ng/mL rcSCF for 24 h prior to collection. Protein lysates were generated, separated by SDS-PAGE and Western blotting for WWOX and β-actin was performed. **d** Fresh frozen canine MCT tissues and **e** malignant canine (BR and C2) and mouse (C57 and P815) mast cell lines were processed for protein lysates. Protein was separated by SDS-PAGE and Western blotting for WWOX and β-actin was performed. Full-length blots are presented in Supplementary Fig. 1. Three independent experiments were performed and all reactions were performed in triplicate. The experiments were repeated 3 times in the cell lines and twice for normal canine BMCMCs

The presence of genetic alterations in WWOX, largely due to loss of heterozygosity, and its reduced expression has been observed in a number of human cancers [42, 43]. Moreover, loss or reduction of WWOX expression corresponds to a worse clinical prognosis in many cancers. To provide an initial assessment of WWOX expression in canine MCT tissues and canine mast cell lines, we first analyzed WWOX transcript levels in primary MCTs and normal canine bone marrow-cultured mast cells (BMCMCs) by qRT-PCR. As shown in Fig. 1a, normal canine BMCMCs exhibited significantly higher levels of WWOX transcript compared to primary canine MCTs. We next evaluated canine malignant mast cell lines (BR and C2) and normal canine BMCMCs for evidence of altered WWOX expression. Concordant with our data generated in primary MCT tissues, RT-qPCR confirmed that WWOX expression is substantially decreased in canine mastocytoma cell lines when compared with normal canine BMCMCs (Fig. 1b).

Canine BMCMCs possess phenotypic and functional properties similar to those described of mast cells directly isolated from canine skin; however, BMCMCs are dependent on recombinant canine stem cell factor (rcSCF) for survival in vitro [44]. In contrast, malignant canine mast cell lines (BR and C2) readily survive and proliferate in standard cell culture conditions independent of rcSCF stimulation. To assess whether culture conditions affected the status of WWOX, the canine BR and C2 mast cell lines were grown in the presence or absence of rcSCF and again evaluated for changes in WWOX protein expression. The basal levels of WWOX remained unchanged despite variation in culture conditions in both canine mastocytoma cell lines evaluated (Fig. 1c).

Additionally, Western blotting of protein lysates derived from fresh frozen canine MCT tissue samples revealed low or non-detectable levels of WWOX protein in 3 of the 5 tumors (Fig. 1d). Although levels of WWOX expression varied among tumor samples included in this analysis, this may be attributed, in part to differences in the heterogeneity of tumor stroma, presence other inflammatory cells within the tumor microenvironment, and/or normal structures within the epidermis and dermis [45]. To assess whether WWOX is aberrantly expressed in malignant canine (BR and C2) and murine (C57 and P815) mast cell lines, Western blotting was performed. As shown in Fig. 1e, the canine BR cells and mouse C57 and P815 cells demonstrated low basal levels of WWOX when compared to canine C2 cells. Interestingly, the C2 mast cell line exhibited relatively high expression of WWOX at both the transcript and protein level compared to the other cell lines; however, WWOX transcript levels were still substantially lower in the C2 mast cell line when compared to normal canine BMCMCs. Collectively, these findings demonstrate that WWOX expression is reduced or attenuated in a subset of primary canine MCTs and mouse and canine mast cell lines, but not in normal canine BMCMCs.

### Generation of canine mast cell lines expressing WWOX lentiviral constructs or shRNAs targeting WWOX

**Fig. 2:**
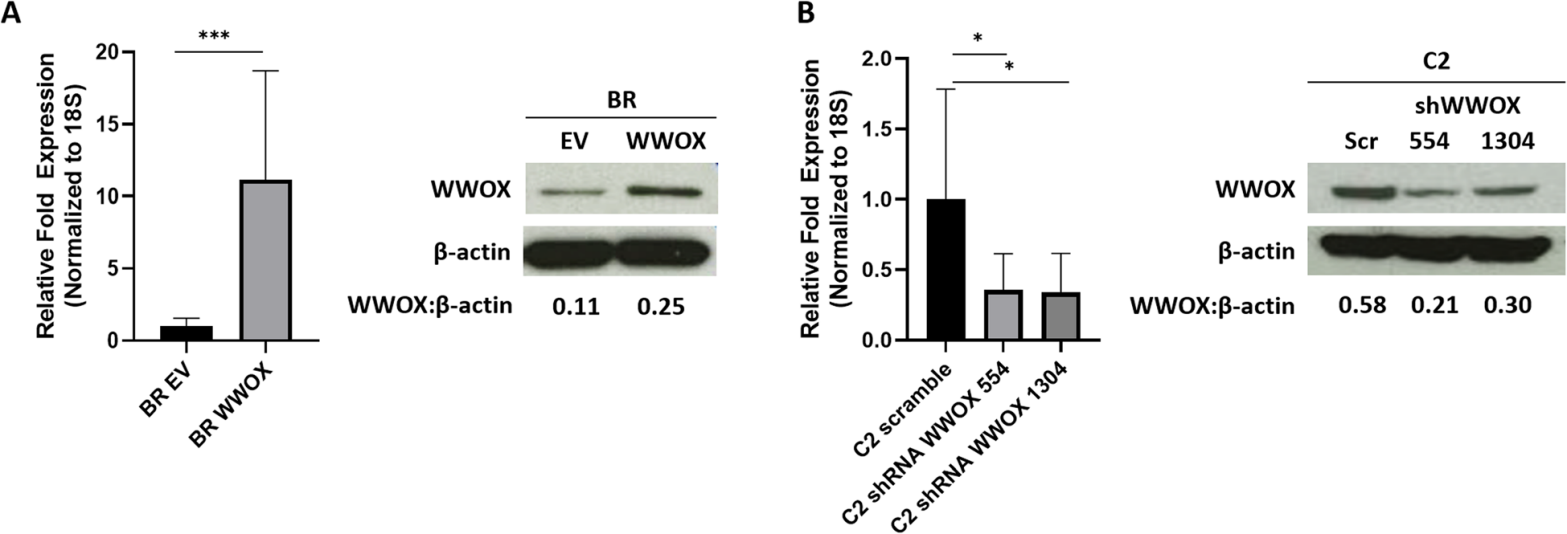
Generation of canine mast cell lines expressing WWOX lentiviral constructs or shRNAs targeting WWOX. **a** Full-length canine *WWOX* cDNA was PCR amplified from canine testes and cloned into the pCDH-copGFP lentiviral expression plasmid (Systems Biosciences). Canine BR cells transduced with empty control vector (EV) or WWOX lentivirus were sorted 72 h post-transduction to greater than 95% purity based on GFP expression. WWOX expression was assessed by qRT-PCR and Western blotting in empty vector or WWOX-expressing BR cells. **b** Canine C2 cells transduced with either scramble (Scr) control vector or pGreenPuro-shWWOX-copGFP lentiviral constructs (shWWOX-554 or shWWOX-1304) underwent FACs-mediated cell sorting based on GFP expression 72 h post-transduction. RNA and protein lysates were generated and qRT-PCR and Western blotting for WWOX and β-actin was performed to confirm efficiency of WWOX knockdown. Full-length blots are presented in Supplementary Fig. 1. Gene expression was calculated using the ΔCt method and all samples were normalized to 18S. Data represent mean +/− SD of three independent experiments and representative blots from three experimental replicates are shown. (Bars: SD. Statistical analysis: one-way ANOVA, *p ≤ 0.05, ***p ≤ 0.001)

To investigate the functional consequences of WWOX dysregulation in malignant canine mast cell lines, the canine BR mast cell line that exhibits low basal levels of WWOX was transduced with a full-length canine WWOX cDNA lentiviral expression vector. Stably transduced GFP + cells were sorted and WWOX overexpression was validated using qRT-PCR and Western blotting (Fig. 2a).

Reciprocal experiments were performed in the canine C2 mast cell line which expresses relatively high levels of WWOX. We designed several lentiviral-shRNAs targeting canine WWOX (shWWOX-554 and shWWOX-1304) to determine the impact of WWOX knockdown on the behavior of C2 mast cells in vitro. As shown in Fig. 2b, WWOX protein and transcript expression was significantly reduced in C2 cells transduced with WWOX-targeted shRNAs as evidenced by Western blotting and qRT-PCR.

### WWOX does not influence cell proliferation and viability in malignant canine mast cell lines

**Fig. 3:**
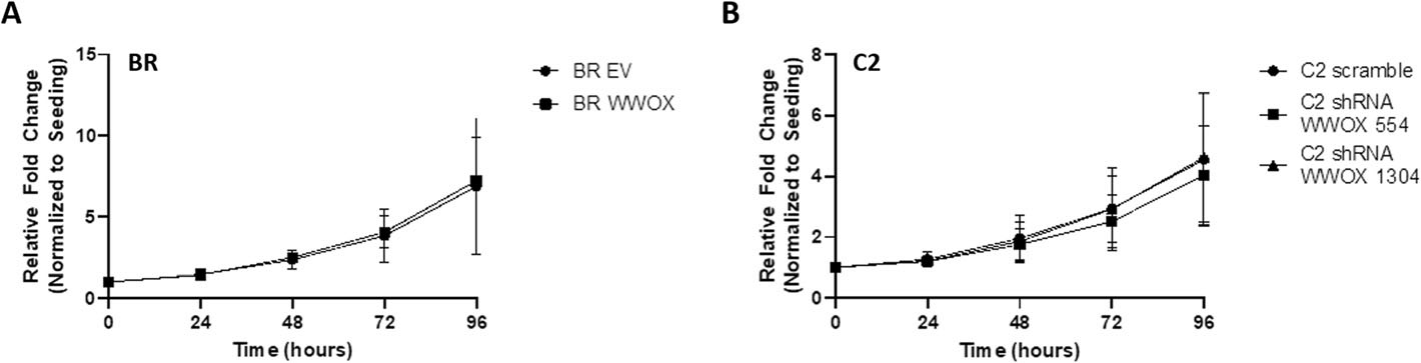
WWOX does not influence cell proliferation and viability in malignant canine mast cell lines. **a** Canine BR cells were transduced with empty control vector (EV) or WWOX lentiviral constructs and **b** C2 cells were transduced with scramble control or shRNA lentiviral constructs targeting WWOX (shWWOX-554 and shWWOX-1304). BR and C2 cells (5 × 10^4^ cell/well) were seeded in 96-well plates and cell viability was assessed at 0, 24, 48, 72, or 96 h using the MTT assay. All experiments were performed in triplicate and data represent the mean +/− SD of three independent experiments

Prior studies have shown that ectopic expression of WWOX through various methods such as adenovirus-delivered WWOX cDNA (Ad-WWOX) or treatment with the DNA methyltransferase inhibitor,, 5-aza-2′-deoxycytidine to activate the endogenous WWOX gene results in decreased cell growth and viability in human breast and lung cancer-derived cells in vitro and in vivo [46, 47]. To further assess the impact of WWOX on canine mast cell growth, BR cells expressing control or WWOX lentiviral constructs or C2 mast cells expressing scramble or shRNAs targeting WWOX (shWWOX-554 and shWWOX-1304) were cultured for 24, 48, 72, and 96 h and mast cell proliferation and viability was evaluated using a standard MTT colorimetric assay. In contrast to data generated in human carcinoma cell lines, enforced expression of WWOX or shRNA-mediated WWOX knockdown had no significant effect on mast cell proliferation and viability in the cell lines evaluated (Fig. 3a, b).

### WWOX-deficient C2 mast cells exhibit increased clonogenic survival of ionizing radiation-induced double strand breaks

**Fig. 4:**
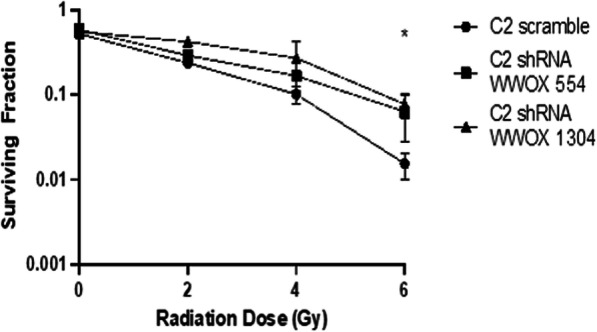
Impact of WWOX on clonogenic survival and cell viability in malignant canine mast cell lines. **a** Canine C2 cells expressing scramble control or shRNA lentiviral constructs targeting WWOX (shWWOX-554 and shWWOX-1304) were treated with increasing doses of ionizing radiation and plated in 6-well dishes in methylcellulose media until formation of visible colonies. Cells were then fixed and stained with crystal violet and colonies greater than 50 cells were counted. After counting colonies, plating efficiency and survival fraction were calculated. Plating efficiency was defined as the number of colonies formed divided by the number of cells seeded in untreated (0 Gy) control groups. Surviving fraction was defined as the number of colonies formed divided by the number of cells seeded in irradiated groups, normalized to the plating efficiency. Experiments were repeated three times and average of results are shown. All experiments were performed in triplicate and data represent the mean +/− SD of three independent experiments (Statistical analysis: one-way ANOVA, *p ≤ 0.05)

Genomic instability is a major risk factor for cancer development and progression. With respect to canine MCTs, genomic copy number profiles of tumors of different histological grades demonstrate a stepwise accumulation of numerical DNA copy number aberrations (CNAs) as histological grade increases, suggesting that these genomic alterations contribute to genomic instability and progression of canine MCTs. Interestingly, four CNA markers (loss of CFA 5:37.8 Mb, gain of CFA 31:16.7 Mb, loss of CFA 20:31.6 Mb, and gain of CFA 20:46.4 Mb) were found to discriminate low-risk from high-risk MCTs with a sensitivity of 78–94% and specificity of 88–93% [14]. Of note, CNAs predictive of aggressive MCT phenotypes include genomic loss of CFA 5 (− 37.8 Mb) a region harboring the canine *WWOX* allele (NCBI Ref Sequence XM_847530.5, chr5: 72,299,157-73,243,633), highlighting the possibility that disruption of *WWOX* may be a key molecular alteration in aggressive MCTs. Furthermore, recent evidence supports a critical role for the tumor suppressor WWOX in DNA damage response and maintenance of genomic stability, in part, through its association with ataxia telangiectasia-mutated (ATM) following double strand DNA (dsDNA) breaks [39, 48]. Given the correlation between WWOX dysregulation and DNA damage repair signaling, we sought to determine whether inhibition of WWOX in canine mast cell lines affected their capacity to proliferate and survive following dsDNA damage. Canine C2 mast cells expressing scramble or shRNA constructs targeting WWOX were treated with increasing doses of ionizing radiation and clonogenic survival was assessed via standard colony formation assay. Radiation administered at doses ranging from 2 to 6 Gy demonstrated a dose dependent reduction in clonogenic survival, as expected, and genetic knockdown of WWOX significantly increased clonogenic survival of C2 mast cells at higher radiation doses (6 Gy *p* = 0.0416, Fig. 4). C2 mast cells expressing WWOX shRNAs showed a trend towards increased clonogenic survival at doses of 4 Gy, but this difference did not reach statistical significance (*p* = 0.1200). In WWOX-deficient C2 mast cells, the shape of the survival curve had broader shoulder compared to the scramble control cells, providing support for the notion that WWOX attenuation may diminish sensitivity to ionizing radiation in this cell line.

Similar colony formation assay experiments were performed with the BR mast cell line; however, no colony growth was observed in the presence of methylcellulose-based media (data not shown), and thus the effect of WWOX overexpression on clonogenic survival of these cells could not be determined using this assay. Alternatively, we evaluated BR cell viability at multiple time points using an MTT assay as a means to assess cell proliferation following ionizing radiation treatment. While treated BR cells a demonstrated dose-dependent decrease in cell viability, no significant difference in cell viability and proliferation was found in BR cells expressing control vector or overexpressing WWOX (data not shown). The lack of observed differences in cell viability indicates that restoring WWOX does not significantly impact sensitivity to ionizing radiation in this cell line.

### Immunohistochemical expression of WWOX in primary canine MCT samples

To more globally assess the prevalence of mast cell-specific WWOX expression in primary canine MCT samples, a tissue microarray was constructed from 45 archived canine MCT surgical biopsies (*N* = 26) and paired normal skin samples (*N* = 19) and immunohistochemical (IHC) staining for WWOX was performed. All tumors were graded using the 2-tier grading system and IHC scoring was performed by two blinded board-certified veterinary pathologists using the specifications outlined in Table 1. 7 of the 26 MCT samples were considered to have inadequate tumor sample and were excluded from analysis.

**Table 1:** Scoring criteria for tumor microarray

Code Assigned	Criteria for Stain Strength	Code Assigned	Percent Cells Labeled
0	No Labeling	0	0% labeling
1	Mild cytoplasmic labeling	1	1–25% labeled
2	Moderate cytoplasmic labeling	2	26–50% labeled
3	Strong cytoplasmic labeling	3	51–75% labeled
		4	76–100% labeled

**Fig. 5:**
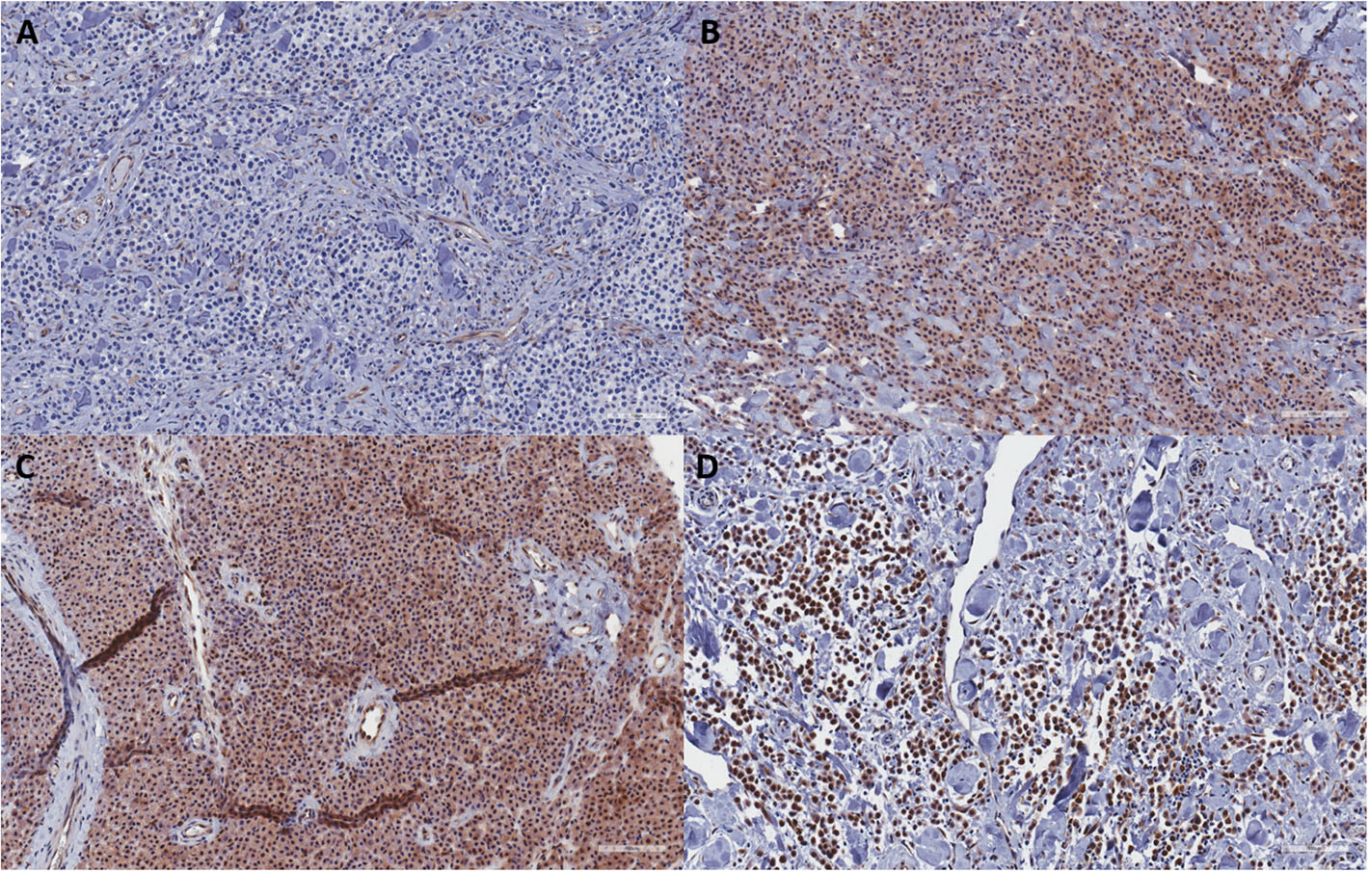
Immunohistochemistry for WWOX in primary canine mast cell tumor samples. Immunohistochemistry was performed for WWOX using a tissue microarray of primary MCTs and paired normal skin biopsies. Signal intensity and percent positivity were scored. Examples of no **(a)**, mild **(b)**, moderate **(c)**, and strong **(d)** stain intensity are shown (20X)

Signal intensity and percent positivity were scored 1–3 or 1–4, respectively, and averaged between scorers. Representative examples of no, mild, moderate, and strong signal intensity are shown in Figs. 5a-d. Non-neoplastic mast cells present in paired normal skin biopsies were identified based on cellular morphology and localization near perivascular regions and demonstrated mild cytoplasmic staining. Additional background labeling in normal endothelium and epithelium was noted in both normal and tumor samples, and thus not counted in primary tumor assessment.

**Fig. 6:**
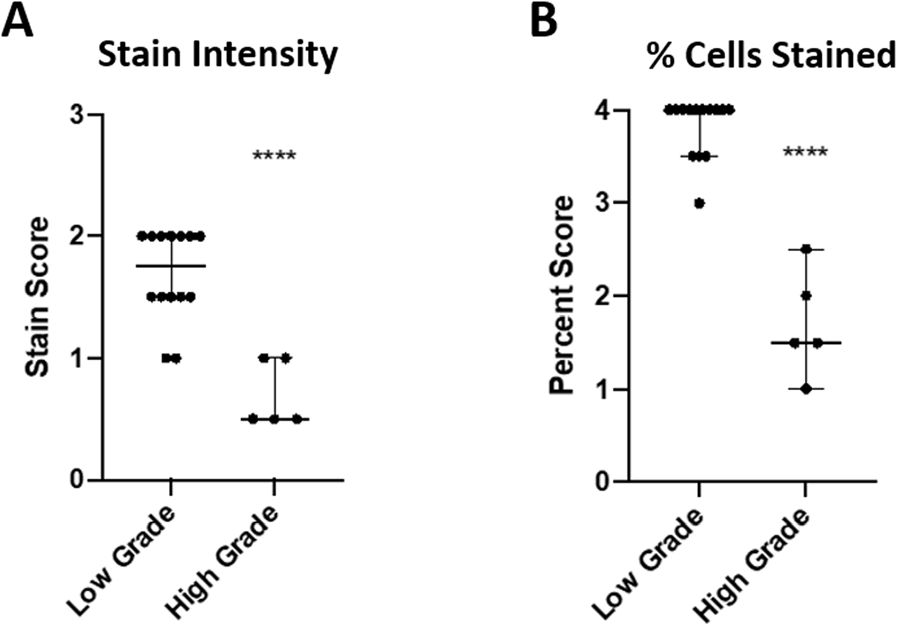
WWOX expression in canine MCTs by immunohistochemistry. Primary MCTs were graded using the two-tiered histologic grading scheme (*N* = 14 low grade, *N* = 5 high grade) and immunohistochemical staining for WWWOX was performed. Tumors were scored blindly by two board-certified veterinary pathologists using the specifications outlined in Table 1. Results were averaged between raters for comparison based on tumor grade. **a** Degree of staining intensity negatively correlated with histologic grade with high-grade MCTs exhibiting significantly lower stain intensity. **b** The percent of neoplastic cells staining was significantly decreased in high-grade MCTs as compared to low grade tumors. Weighted Kappa scores were determined and there was fair interrater agreement for both staining intensity and percent of cells stained (K = 0.264 and 0.273, respectively; statistical analysis: one-way ANOVA, ****p ≤ 0.0001)

The degree of WWOX staining intensity was found to negatively correlate with histologic grade such that mast cells present in high-grade tumors (*N* = 5) showed significantly lower staining intensity compared to low-grade tumors (*N* = 14) (Fig. 6a). We found that the percent of neoplastic cells staining for WWOX was also significantly decreased in high-grade MCTs, supporting the idea that loss of WWOX may correlate with a more aggressive tumor phenotype (Fig. 6b). There was fair interrater agreement for both staining intensity and percent of cells stained (K = 0.264 and 0.273, respectively).

## Discussion

The molecular characteristics and chromosomal location of canine fragile sites have not been intensively studied; however, recent genomic profiling data demonstrate that canine MCTs exhibit a broad range of genome-wide copy number aberrations (CNAs) and that the frequency of CNAs increases with histological grade [14]. These data support the notion that genomic instability is a defining feature in high-risk canine MCTs and suggest that loss or gain of genes within copy number aberrant regions may contribute to a more aggressive tumor phenotype. Notably, recurrent CNAs associated with aggressive MCT behavior include genomic loss of CFA 5 (− 37.8 Mb) a region harboring the canine *WWOX* allele. Concordantly, we found that primary canine MCTs and malignant mast cell lines express low basal levels of WWOX transcript compared to that found in normal canine BMCMCs. Low levels of WWOX were detected by Western blotting in malignant canine and mouse mast cell lines evaluated and this was independent of culture conditions and rcSCF stimulation. Similar to our findings in mast cell lines, WWOX protein expression was low to absent in a subset of fresh frozen MCTs derived from canine patients. These findings support previous studies demonstrating that WWOX is frequently attenuated or lost in human malignancies and suggest its loss may be involved the neoplastic transformation of canine mast cells [17, 20, 25, 49].

To assess the prevalence of basal WWOX expression in primary canine MCT tissue samples, we constructed a TMA from archival formalin-fixed paraffin embedded tissue samples and performed IHC for WWOX. We analyzed WWOX expression levels in primary canine MCTs and found that the intensity of WWOX staining and percentage of stained neoplastic cells was significantly lower in high-grade MCTs. The limited number of primary MCTs evaluated in the current study precludes us from drawing conclusions about the expression of WWOX in high-risk MCTs. However, the observation that WWOX expression is attenuated in high-grade MCT provides support for the notion that loss of WWOX may correlate with the degree of mast cell differentiation and the acquisition of more a more aggressive biological behavior. Given the lack of complete patient information and follow-up accompanying the archival canine tumor samples, it was not possible to determine the prognostic significance of WWOX immunoreactivity in canine MCTs. Nevertheless, the finding that WWOX expression is frequently decreased in histologically high-grade tumors merits further investigation as to the role of WWOX in promoting an aggressive tumor phenotype.

To define the functional consequences of altered WWOX expression on mast cell behavior, we generated canine BR mast cells stably overexpressing WWOX or inhibited WWOX function in the canine C2 cell line using shRNA techniques. Interestingly, enforced WWOX expression or WWOX knockdown had no effect on cell proliferation in the BR or C2 mast cell lines. Additionally, overexpression of WWOX in the canine BR mast cell lines did not significantly alter cell viability following treatment with ionizing radiation. In contrast to our study, numerous studies have shown that WWOX directly influences cell viability and survival in normal and malignant epithelial cells in vitro and in vivo in mouse xenograft tumor models [21]. These observed differences may be attributed, in part, due to the versatile nature of WWOX and its ability to interact with different proteins in multiple cellular pathways. Structurally, WWOX’s tandem WW domains provide the protein with the ability to bind a wide range of different partners resulting in cytoplasmic sequestration and inactivation [17]. As such, the phenotypic consequences of altered WWOX expression on cell behavior may be largely influenced by the contextual nature of WWOX and the expression of putative binding partners in distinct cell types/tissues. Furthermore, differences in the capacity of WWOX to inhibit cell viability has been observed in different cancer cell lines. For example, data generated in human ovarian cancer cell lines deficient in WWOX demonstrate that restoration of WWOX abolishes in vivo tumorigenicity in nude mice and promotes apoptosis in suspension cultures in vitro but does not alter cell growth rate. In ovarian carcinoma cell lines, WWOX was found to act as a tumor suppressor by modulating the interaction of tumor cell integrin-α3 with extracellular matrix components (fibronectin) and promoting apoptosis in detached cells [50].

In the current study, the canine BR mast cell line exhibited poor overall survival and colony formation using methycellulose-based media; as such, the effect of WWOX restoration on clonogenic survival following IR could not be fully assessed using a standard colony formation assay in this cell line. This cell line-intrinsic finding may reflect potential differences in cell surface protein expression that impacts the formation of clonogenic colonies in the BR cell line, further demonstrating the complicated genetic heterogeneity known in these tumors [14, 51]. Although BR cell viability was evaluated via MTT assays, no differences in cellular survival following IR treatment was observed in BR cells overexpressing WWOX. However, this methodology analyzes survival of both clonogenic and non-clonogenic cell populations and as such, the limitations of this assay may preclude definitive determination of the effect of WWOX restoration on clonogenic survival in the BR cell line. Additionally, the MTT assay is influenced by cell density, and thus has limitations on length of experiment duration when compared to standard clonogenic colony formation assays. Thus, the MTT assay when used in the context of radiation survival may not capture an accurate representation of the surviving fraction when compared to the colony formation assay due to radiation induced alterations in growth rate, and thus doubling time [52]. Further evaluation of the transcriptional and proteomic profiles in response to WWOX expression in the BR and C2 cell lines may provide a more detailed understanding of the signaling pathways directly altered by WWOX that underlie the observed phenotypic differences in these cell lines.

One of the more intriguing findings in this study was that inhibition of WWOX enhanced clonogenic survival in the C2 mast cell line following radiation-induced double strand DNA (dsDNA) breaks. This finding was independent of cell viability and supports the idea that WWOX may play a role in mediating the DNA damage repair in response to dsDNA breaks. Prior studies have demonstrated that WWOX expression is increased following single- and double-stranded DNA breaks and it is recruited to sites of DNA damage. The recruitment and activation of WWOX facilitates DNA damage repair mediated in part, through its direct interaction with ATM and indirectly, through ATR [38, 48]. Loss of WWOX directly impairs DNA damage repair signaling and cell cycle checkpoint activation, thereby allowing for the accumulation of unrepaired DNA breaks. As such, WWOX has been proposed to function as genome caretaker. This is supported by experimental data suggesting that reduced WWOX expression, a common occurrence in cancers, dysregulates dsDNA break repair, and enables resistance to dsDNA damaging agents [53]. Our findings suggest that WWOX dysregulation may play a role in mediating the DNA damage repair response in malignant canine mast cells. Given that ionizing radiation is commonly used in the treatment of non-resectable, aggressive canine MCTs [48, 54], our finding that WWOW knockdown enhances clonogenic survival in the C2 mast cell line may have broader implications with respect to whether WWOX expression levels in MCTs could be an important predictor of response to radiation.

The present study investigated the impact of WWOX expression on certain aspects of cell behavior; however, the molecular factors by which WWOX influences clonogenic survival in mast cells requires further investigation. Furthermore, inhibition of WWOX binding partners that mediate this phenotype in canine mast cell lines would provide further convincing evidence of the importance of WWOX in the DNA damage repair response. As such, identifying proteins altered by WWOX that enhance clonogenic survival and validating these targets in canine mast cell lines/tumors represents an area of ongoing investigation.

## Conclusions

Our data demonstrate that WWOX expression is significantly decreased in primary canine MCT tissues and malignant canine mast cell lines compared to normal canine BMCMCs. In addition, the intensity and percentage of neoplastic cells staining for WWOX via IHC was negatively associated with histologic grade in canine MCTs, further supporting the notion that loss of WWOX contributes to the aggressive biological behavior of some canine MCTs. In the C2 canine mast cell line, shRNA-mediated WWOX knockdown enhanced clonogenic survival in response to ionizing radiation-induced dsDNA breaks, implying that WWOX may play a direct role in mediating the DNA damage repair response in malignant mast cells. This work serves as the foundation for future work to dissect the molecular mechanisms by which WWOX dysregulation contributes to the aggressive biological behavior of malignant canine mast cells, with the ultimate goal of identifying new targets for therapeutic intervention in this disease.

## Methods

### Tumor microarray construction and immunohistochemistry

Primary cutaneous mast cell tumor (MCT) tissue samples were collected from clinical cases that presented to The Ohio State University Veterinary Medical Center (OSU-VMC). Consent for tissue collection was obtained from all owners in accordance with an approved IACUC protocol (2010A0015) and collected by the OSU-VMC Blue Buffalo Clinical Trials Office and Veterinary Clinical Research Shared Resource. A total of 26 primary MCTs with 19 paired normal skin biopsies were identified. Surgical and post-mortem collected tumor samples were placed in formalin and processed for routine paraffin embedding for histopathology. This was a pilot study with the intent to characterize the expression of WWOX in primary canine MCTs and malignant cell lines, so no power calculation was made.

Representative areas of tumor tissue and skin containing vasculature and normal mast cells were identified on hematoxylin and eosin (HE) stained sections by a single, board-certified veterinary pathologist (RJ) and graded using the Patnaik and Kiupel grading schemes. 2.0 mm core samples were extracted from the corresponding areas on 45 paraffin embedded tissue blocks and inserted into predetermined sites on the tissue microarray (TMA) recipient block. Immunohistochemical staining was performed on all MCT and normal skin samples for WWOX using rabbit anti-WWOX antiserum (generously provided by Dr. Kay Huebner, The Ohio State University, Columbus, OH, USA). This antiserum detects endogenous and recombinant human and murine WWOX amino acid residues 12–94 containing both of the WW domains. Human and canine WWOX exhibit 94.4% similarity at the protein level and the region corresponding to WWOX amino acids 12–94 shares 96% sequence homology [55, 56]. Normal canine testes tissue served as a positive control. Negative controls consisted of irrelevant isotype matched antibody at matched dilutions. Both the construction of the TMA bloc and immunohistochemical staining were performed by the OSU-CVM Comparative Pathology and Mouse Phenotyping Shared Resource.

Slides were evaluated by light microscopy to assess WWOX immunoreactivity. Overall WWOX signal intensity of normal and malignant mast cells was subjectively scored from 0 to 3 (0 = none to weak, 1 = mild, 2 = moderate, 3 = strong) by two boarded veterinary pathologists (RJ, CP) using the specifications detailed in Table 1. The percentage of positive cells was also estimated and scored from 0 to 4 (0 = no staining, 1 = 1–25%, 2 = 26–50%, 3 = 51–75%, 4 = > 75%). Total scores were averaged between raters for comparison based on tumor grade.

### Cell lines and reagents

The canine mastocytoma cell line BR (activating point mutation L575P in the JM domain of *KIT)* and C2 (*KIT* ITD mutation in the JM domain) were generously provided by Dr. Warren Gold (Cardiovascular Research Institute, University of California – San Francisco, CA, USA) and murine cell lines P815 (D814V *KIT* mutation) and C57 (wild-type *KIT*) were generously provided by Dr. Stephen Galli (Stanford University, Stanford, CA, USA). The cells were maintained in Roswell Park Memorial Institute medium (RPMI) 1640 (Gibco® Life technologies, Grand Island, NY, USA), supplemented with 10% fetal bovine serum (FBS) (Catalog # 12483–020, Gibco® Life technologies), Antibiotic-Antimycotic (Cat # 15240–062, Gibco® Life technologies), GlutaMAX™ (Cat # 1963762, Gibco® Life technologies), non-essential amino acids, sodium pyruvate, and HEPES (4-(2-dydroxethyl)-1-piperazineethanesulfonic acid) at 37 °C, supplemented with 5% CO_2_ (media supplements from Gibco®). Canine bone marrow cultured mast cells (cBMCMCs) were generated from 2 dogs and maintained in Stemline (Sigma-Aldrich, St. Louis, MO, USA) medium supplemented with recombinant canine stem cell factor (R & D Systems, Minneapolis, MN, USA) as previously described [57].

### RNA isolation, cDNA synthesis, and qRT- PCR

Total RNA was extracted from mast cell lines or primary MCTs using the RNeasy Mini Kit (QIAGEN, Hilden, Germany) according to the manufacturer’s instructions. cDNA was made from 500 ng total RNA using Superscript III (Invitrogen ThermoFisher Scientific, Waltham, MA, USA). Primers designed and utilized for canine WWOX and 18S endogenous control are listed in Table 2. An annealing temperature of 60 °C was used for all reactions. Standard PCR was performed with all primer sets and amplicon length was verified through agarose gel electrophoresis and visualization of PCR products using the Alpha Imager system (Alpha Innotech Corp, San Leandro, CA).

**Table 2:** Primers for quantitative reverse transcriptase polymerase chain reaction

Primers	Primer sequence
K9 RT WwoxF	5′-TCCTCCGAGTCCCATAGATTC −3′
K9 RT WwoxR	5′-CGGCAGCAGTTGTTGAAGTA-3’
18S574F	5′-AAATCCTTTAACGAGGATCCATT-3’
18S652R	5′-AATATACGCTATTGGAGCTGGA-3’

Real-time quantitative PCR (RT-qPCR) was performed to measure WWOX transcript expression using the methods described above. qRT-PCR was performed using Applied Biosystem’s StepOne Plus Real-Time PCR system (Applied Biosystems ThermoFisher Scientific). Canine WWOX and 18S were detected using Fast SYBR Green PCR master mix according to the manufacturer’s instructions. Data normalization was performed relative to 18S internal control. Experiments were repeated three times using samples in triplicate and included non-template controls for each gene. Relative gene expression for all RT-qPCR data was calculated using the comparative threshold cycle method [58].

### Immunoblotting

To assess the relative expression of WWOX, protein lysates were generated from primary MCT tissues or mast cell lines. Briefly, frozen tumor samples were pulverized using a frozen mortar and pestle. The resulting powder was resuspended in liquid nitrogen and transferred to 1.5 mL microcentrifuge tube. Tumor samples and mastocytoma cell lines were washed twice with 1X Dulbecco’s phosphate-buffered saline (DPBS, Gibco) and resuspended in complete lysis buffer consisting of 20 mM Tris-HCl pH 8.0, 137 nM NaCl, 10% glycerol, 1% IPEGAL CA-630, 10 mM ethylenediaminetetraacetic acid (EDTA), 1 mg/mL aprotinin, 1 mg/mL pepstatin A, 1 mM phenylmethylsulphonyl fluoride, 1 mM sodium orthovanadate, and 10 mM sodium fluoride. Samples were rocked for 1 h at 4 °C, centrifuged for 15 min at 14,000 RPM at 4 °C, and supernatants collected. Bradford protein quantification assay was performed on the extracts using BioRad Reagent (Cat #5000006, BioRad, Hercules, CA, USA) according to manufacturer’s instructions. Equal amounts of total protein were separated on 12% SDS-PAGE gels (Cat #4561093, Bio-Rad) and transferred onto PVDF membranes. Membranes were blocked in TBS-T containing 3% non-fat dry milk for 1 h and incubated overnight with polyclonal rabbit anti-WWOX antibody (Cat #PA5–17237, Invitrogen ThermoFisher Scientific). The membranes were incubated with appropriate horseradish peroxidase linked secondary antibody (Cat# 7074S, Cell Signaling, Danvers, MA), washed, and exposed to luminol enhancer Supersignal® West Dura Extended Duration Substrate developer (Cat #34075, ThermoFisher Scientific). Blots were stripped using Restore™ Western Blot Stripping Buffer (Cat #21059, ThermoFisher Scientific), washed, and reprobed for β-actin (Cat #3700S, Cell Signaling, Danvers, MA, USA). Band intensities were calculated using ImageJ software (NIH, Bethesda, MD, USA) and relative intensity of WWOX was determined by dividing by β-actin.

### Recombinant stem cell factor co-culture

To determine the potential effect of stem cell factor (SCF) stimulation on WWOX expression in canine mast cell lines, BR and C2 cells (1 × 10^7^) were cultured in complete media supplemented with 100 ng/mL canine recombinant SCF (Cat #2278SC025, R&D Systems Inc., Minneapolis, MN) or left untreated for 24 h at 37 °C supplemented with 5% CO_2_. Cells were collected, washed twice with 1X DPBS, and immunoblotted to detect WWOX expression was performed as previously described.

### WWOX and shWWOX lentivirus infection

Full-length canine WWOX cDNA was amplified by RT-PCR from normal canine testes and the resultant product was gel purified and sequenced. Canine WWOX cDNA was ligated into the pGEMT plasmid vector (Promega, Madison, WI, USA), and subcloned into the pCDH-CMV-MSC-copGFP lentiviral expression plasmid (Cat #CD511B-1, Systems Biosciences, Palo Alto, CA, USA). Packaging of the lentiviral constructs was performed using the pPACKH1 Lentivector Packaging Kit (Cat #LV500A-1, System Biosciences) according to manufacturer’s instructions. Briefly, 1 × 10^6^ BR cells were incubated overnight in complete medium. The following day, medium was changed to antibiotic-free medium with transfection agent TransDux™ (Cat # LV850A1, Systems Biosciences, Mountain View, CA, USA) and cells were infected with empty control (EV) lentivirus or WWOX lentivirus or TransDux™ alone for 24 h. FACS-mediated sorting based on GFP expression was performed 72 h post-transduction and WWOX overexpression was validated by qRT-PCR and Western blotting analysis.

Stable knock down of WWOX was performed using short hairpin RNA (shRNA) constructs cloned into the pGreenPuro shRNA cloning lentivector (Cat # SI505A-1, System Biosciences) and high-titer lentiviral stocks were generated as described above. Briefly, 1 × 10^4^ C2 cells were plated and left overnight in complete medium. The following day, medium was replaced with serum-free medium and target cells were infected with transfection agent TransDux™ (Systems Biosciences) and either pGreenPuro-Scramble or pGreenPuro-shWWOX virus or TransDux™ alone for 24 h. FACS-mediated sorting based on GFP expression was performed 72 h post-transduction. Cells were collected and processed for qRT-PCR and Western blotting as described below to detect levels of WWOX and efficiency of knock down. Sequences of template canine DNA were as follows: pGreenPuro-shWWOX-554 (5′-CCCTATGGATGGGAACAAGAA-3′) and pGreenPuro-shWWOX-1304 (5′-ACATGATGTACTCCTCCATTC-3′).

### Assessment of cell viability and cell proliferation

To determine the effects of WWOX dysregulation on mast cell viability, BR cells (5 × 10^3^) transduced with EV control or WWOX lentiviral constructs and C2 cells (5 × 10^3^) expressing scramble, shWWOX-554 or shWWOX-1304 constructs were seeded in complete medium in quadruplicate in 96-well plates and incubated for 24, 48, 72 or 96 h. To determine the influence of WWOX expression on mast cell viability following treatment with DNA damaging agents, BR cells (5 × 10^3^) transduced with EV control or WWOX lentiviral constructs were treated with increasing doses (0, 2, or 4 Gy) of ionizing radiation (IR) using a Siemen’s ONCOR linear accelerator and incubated for 24, 48, 72, or 96 h. 10 μL of MTT reagent (Cat #11465007001, Roche, Mannheim, Germany) was added to each well and cells were incubated for an additional 4 h under normal culture conditions. Following incubation, 100 uL lysis buffer was added and the fluorescence was measured using a SpectraMax M2 microplate reader (Molecular Devices, Sunnyvale, CA, USA) with excitation at 570 nm and emission 670 nm filters. Cell viability and proliferation was calculated as the fold change of absorbance from 0 h control cells. Each experiment was repeated three times using samples in quadruplicate.

### Clonogenic survival assay

BR cells expressing EV or WWOX constructs and C2 cells expressing scramble, shWWOX-554 or shWWOX-1304 constructs were irradiated with increasing doses (0, 2, 4, or 6 Gy) of ionizing radiation at ambient temperature and pressure utilizing a linear accelerator (ONCOR System, Siemens AG, Muenchen, Germany, EU). Cell culture flasks (75 cm^3^ with 10 cm^3^ complete media with water-equivalent height of 4 mm) were placed above a solid water-equivalent plate (12 mm) and irradiated from below with the gantry at 180°. The dose distribution for this set up was verified by a medical physicist. Control (0 Gy treatment) cell culture plates were transported to the radiation therapy area but kept outside the radiation vault during treatments. After individual flasks were treated with doses of 0, 2, 4, and 6 Gy of radiation, cells were counted and plated to appropriate density based on dose received (Table 3) and resuspended in MethoCult™ (Cat #-4535, STEMCELL Technologies, Vancouver, BC) to a final volume of 1.1 mL per the manufacturer’s instructions. Cells were seeded in triplicate in a 6-well plate in standard culture conditions and the inter-well space supplemented by 8 mL sterile water to maintain humidity. Colony formation was monitored daily and the experiment stopped after 17 days, before the control colonies became confluent. Surviving colonies consisting of > 50 cells were counted using a counting grid, and the plating efficiency and surviving fraction were calculated as previously described [59]. Experiments were performed in triplicate and repeated three times.

**Table 3:** Density of seeding for clonogenic assay

Treatment Dose (Gy)	C2 Cells Plated per Well	BR Cells Plated per Well
0	125	250
2	250	500
4	500	750
6	1000	1000

### Statistics

Experiments were performed in triplicate or quadruplicate and repeated three independent times. Data were presented as mean plus or minus standard deviation. qRT-PCR data was normalized to internal control (18S) and the ∆∆ Ct method was used to compare mRNA expression using Student’s t-test or one-way analysis of variance (ANOVA) [58]. TMA data was analyzed using Student’s t-test and inter-rater reliability was assessed via Cohen’s kappa with Landis & Koch interpretations [60]. Group comparisons in the MTT proliferation assays and clonogenic survival assays were analyzed by one-way ANOVA. Values of *p* < 0.05 were considered statistically significant.

## Supplementary Information

**Additional file 1: Figure S1.** Uncropped Western blots. (Fig. 1C) Canine BR mast cell lines were left untreated (Lane 1) or incubated with 100 ng/mL rcSCF (Lane 2) and canine C2 mast cell lines were left untreated (Lane 3) or incubated with 100 ng/mL rcSCF. Western blotting for WWOX (~ 47 kDa, upper panel) and β-actin (43 kDa, lower panel) was performed. (Fig. 1E) Canine BR (Lane 5) and C2 (Lane 6) cell lines and mouse C57 (Lane 7) and P815 (Lane 8) mast cell lines were processed for protein lysates. Western blotting for WWOX (upper panel) and β-actin (lower panel) was performed. (Fig. 1D) Canine primary mast cell tumors (Lanes 1–5) were probed for WWOX (upper panel) and β-actin (lower panel). (Fig. 2A) Canine BR-empty vector (Lane 1) and BR-WWOX (Lane 2) cell lines were probed for WWOX (upper panel) and β-actin (lower panel). (Fig. 2B) Canine C2-scramble (Lane 1), C2-shWWOX-554 (Lane 2) and C2-shWWOX-1304 (Lane 3) cell lines were probed for WWOX (upper panel) and β-actin (lower panel). Red arrows indicate ~ 47 kDa band or 43 kDa band corresponding to WWOX or β-actin, respectively. L = Protein Ladder. Dashed lines indicated cropped areas presented in main manuscript text.

## Data Availability

The datasets used and/or analysed during the current study are available from the corresponding author on reasonable request.

## Abbreviations

MCT: Mast cell tumor
MST: Median survival time
CNA: Copy number aberrations
BMCMC: Bone-marrow-cultured mast cells
WWOX: WW domain-containing oxidoreductase
HE: Hematoxylin & eosin
TMA: Tumor microarray
RT-qPCR: Real-time quantitative polymerase chain reaction
SCF: Stem cell factor
EV: Empty vector control
IR: Ionizing radiation
ANOVA: Analysis of variance
shRNA: Short-hairpin RNA
CFS: Common fragile site
