# Supplementary figures and images for: Characterization of WWOX expression and function in canine mast cell tumors and malignant mast cell lines
Source: BMC Vet Res. 2020 Oct 31;16:415. doi: 10.1186/s12917-020-02638-3 (PMC7603737; doi:10.1186/s12917-020-02638-3)

Figure 1C Figure 1E

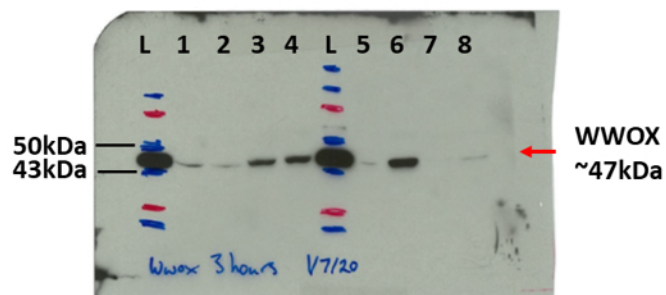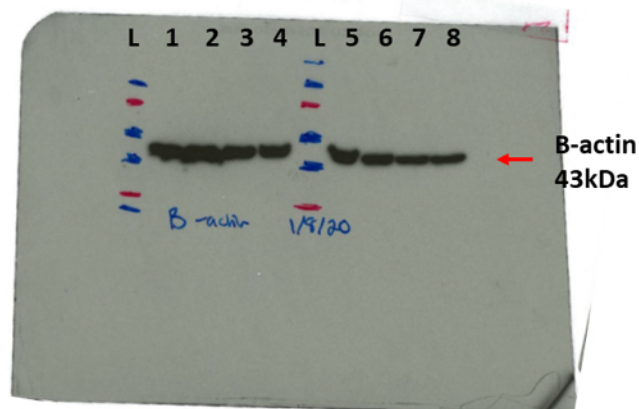

Figure 1D

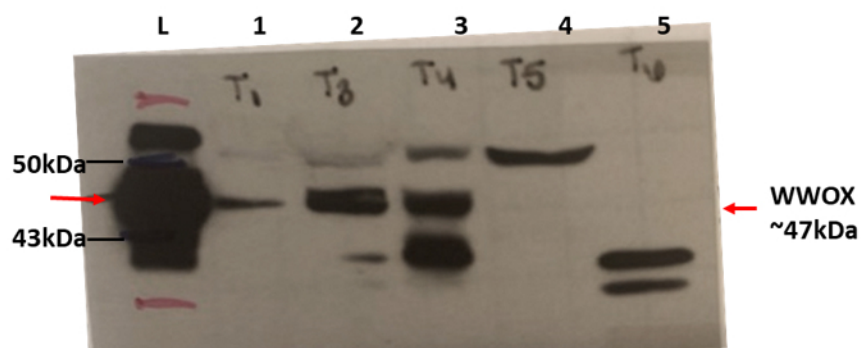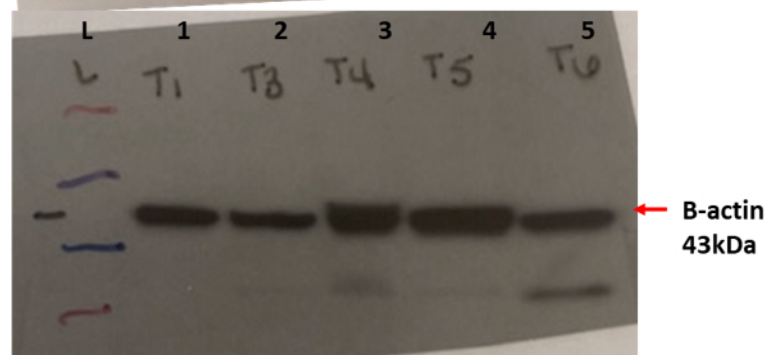

Figure 2A

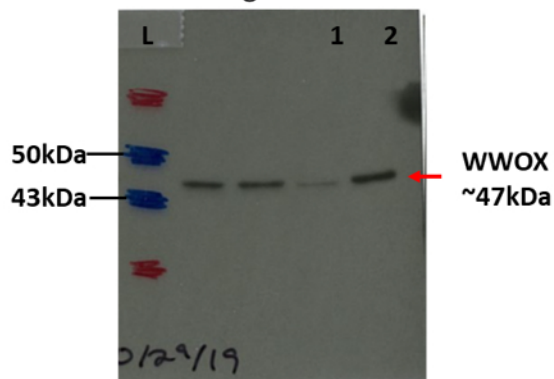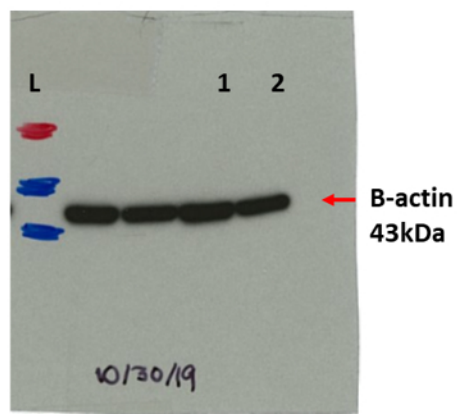

Figure 2B

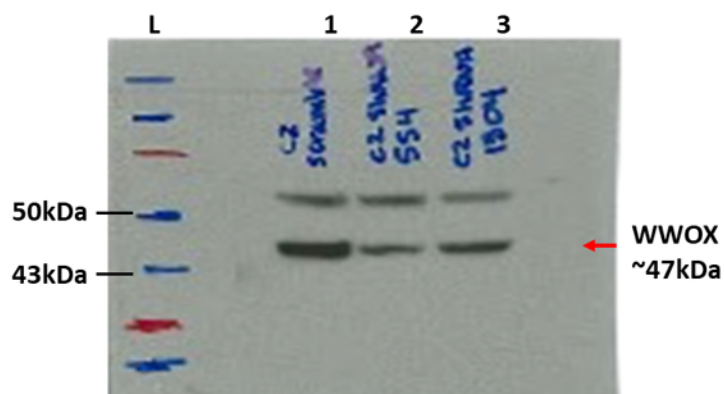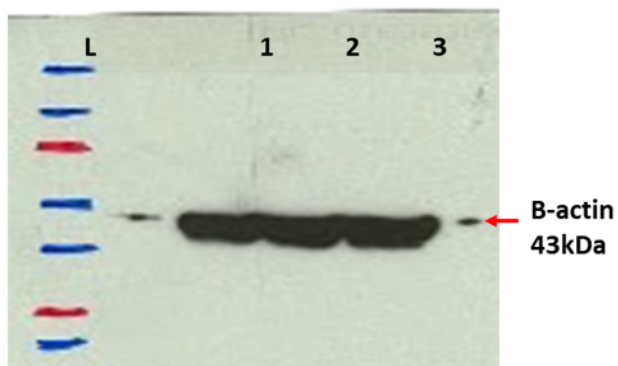

Supplement: Supplementary file 1 — Additional file 1: Figure S1. Uncropped Western blots. (Fig. 1C) Canine BR mast cell lines were left untreated (Lane 1) or incubated with 100 ng/mL rcSCF (Lane 2) and canine C2 mast cell lines were left untreated (Lane 3) or incubated with 100 ng/mL rcSCF. Western blotting for WWOX (~ 47 kDa, upper panel) and β-actin (43 kDa, lower panel) was performed. (Fig. 1E) Canine BR (Lane 5) and C2 (Lane 6) cell lines and mouse C57 (Lane 7) and P815 (Lane 8) mast cell lines were processed for protein lysates. Western blotting for WWOX (upper panel) and β-actin (lower panel) was performed. (Fig. 1D) Canine primary mast cell tumors (Lanes 1–5) were probed for WWOX (upper panel) and β-actin (lower panel). (Fig. 2A) Canine BR-empty vector (Lane 1) and BR-WWOX (Lane 2) cell lines were probed for WWOX (upper panel) and β-actin (lower panel). (Fig. 2B) Canine C2-scramble (Lane 1), C2-shWWOX-554 (Lane 2) and C2-shWWOX-1304 (Lane 3) cell lines were probed for WWOX (upper panel) and β-actin (lower panel). Red arrows indicate ~ 47 kDa band or 43 kDa band corresponding to WWOX or β-actin, respectively. L = Protein Ladder. Dashed lines indicated cropped areas presented in main manuscript text. [file 12917_2020_2638_MOESM1_ESM.pdf]
